# In Situ Stimuli Transfer in Multi‐Environment Shape‐Morphing Hydrogels Based on the Copolymer Between Spiropyran and Acrylic Acid

**DOI:** 10.1002/advs.202416173

**Published:** 2025-03-07

**Authors:** Liwei Wu, Yiming Liu, Wenpei Yang, Zejun Liu, Cuiping Liu, Xiaomin Yuan, Lingling Zhang, Jie Ju, Xi Yao

**Affiliations:** ^1^ Key Lab for Special Functional Materials of Ministry of Education School of Nanoscience and Materials Engineering Henan University Kaifeng Henan 475004 P. R. China

**Keywords:** hydrogel, polyacrylic acid, shape morphing, spiropyran

## Abstract

Smart hydrogels are considered as close mimics to the functions of biological entities. However, the stimuli‐responsive performance of hydrogels is often limited by the slow diffusion process during water exchange with the surrounding environment. Here, a homogenous hydrogel composed of a water‐soluble spiropyran covalently attached to the polyacrylic acid network is reported. This hydrogel demonstrates rapid and reversible shape‐morphing behavior in air, underwater, or in oil. The mechanism involves the reversible protonation of spiropyran triggered by light stimuli. The release/capture of protons regulates the local proton concentration near the carboxyl groups in the polyacrylic acid network, distinguishing it from existing stimuli‐responsiveness based on bulk water diffusion. The environment‐independent shape‐morphing performance of the unique in‐situ stimuli transfer process, resulting in local water transfer amongst parts of a single piece of hydrogel is attributed. Eventually, light‐controlled reversible actuation of the hydrogel is demonstrated, offering exciting possibilities for applications in flexible electronics, and soft actuators/robots.

## Graphical abstract

Poly (acrylic acid‐co‐spiropyran)‐based hydrogel is designed as a photo‐stimuli shape‐morphing material. This hydrogel exhibits rapid and reversible photo‐morphing behavior in various environments, including water, oil, and open air. This behavior is attributed to the light‐triggered protonation of spiropyran, which allows for the in situ transfer of water among different parts of the hydrogel.

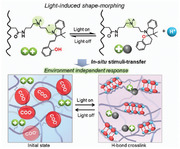

## 1 Introduction

Smart hydrogels based on pH response, thermal response, and other materials have never left the focus of research for our constant needs in sensors,^[^ ^1^, ^2^ ^]^ microfluidics,^[^ ^3^ ^]^ drug delivery,^[^ ^4^, ^5^, ^6^ ^]^ actuators,^[^ ^7^, ^8^, ^9^ ^]^ and soft robotics,^[^ ^10^, ^11^ ^]^ etc. However, their stimuli‐responsive behavior is bound by the slow process of water uptake and release.^[^ ^12^, ^13^ ^]^ Such dependence on water makes it impractical to utilize these materials in speed‐related situations. Moreover, the application of stimuli‐responsive materials is greatly restricted in water‐free environments, such as oil, and in low‐water environments, like air. New attempts for thermo‐responsive hydrogels are being developed to unbound hydrogels from their water environment.^[^ ^14^ ^]^ For example, T. Aida et al. designed a PNIPAm hydrogel that exhibits shape morphing without water exchange to its surroundings. Arrays of electrolyte nanosheets were incorporated into the gel matrix, exerting electrostatic repulsion and activating anisotropic shape‐morphing behavior.^[^ ^15^ ^]^

Similar to thermal‐responsive hydrogels, the pH‐responsive hydrogels undergo massive volume changes based on internal water transfer. However, the pH‐responsiveness is unlimited by the slow thermal transfer process and can undergo substantial volume changes by several hundred times, even when subjected to slight variations in external pH levels. PAAc hydrogel is one of the most important pH‐responsive soft materials.^[^ ^16^, ^17^, ^18^ ^]^ Its response is attributed to the weak polyelectrolyte nature of carboxyl groups.^[^ ^19^, ^20^ ^]^ Much like the aforementioned cases, the response of PAAc hydrogel is governed by the mass transfer of water into or out of the hydrogels, resulting in a slow response rate and a strong reliance on the water‐based pH‐shifting environments.^[^ ^21^ ^]^ The key to changing this situation lies in a new strategy to shift the local pH at the vicinity of carboxyl groups inside the gel network in a new route that effectively avoids the mass diffusion process in/out of the bulk material. For this purpose, it's necessary to utilize some source of stimulus, such as light, electric field, etc., that penetrates through the bulk with no delay.^[^ ^22^, ^23^, ^24^, ^25^ ^]^ Then, such a stimulus should be able to in‐situ shift local pH for the carboxyl groups nearby, triggering their pH responsiveness. Such a mechanism combines the advantages of the two stimuli‐responsive paradigms, achieving rapid, large amplitude, and environmentally‐independent shape morphing function. We refer to this one‐stimulus‐passed‐to‐anther process as the in‐situ stimuli transfer mechanism. A development by D. Diamond et al. created an in situ acidity regulation system consisting of the copolymer among spiropyran, NIPAm, and AAc.^[^ ^26^ ^]^ The isomerization of protonated merocyanine molecules to spiropyran moieties under irradiation releases protons reversibly, leading to significant in‐situ pH regulation of PAAc. This attempt represents a promising step toward achieving an in situ stimuli transfer mechanism, although the mass diffusion of water remains dominant in this case.

**Figure 1:**
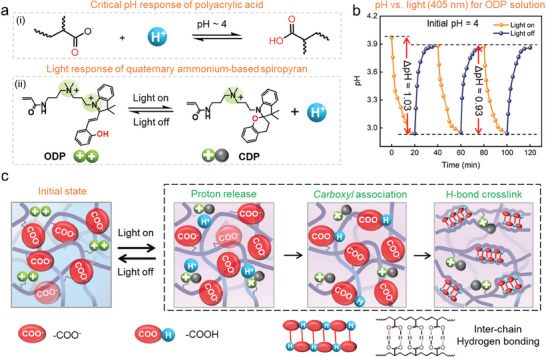
Design of photo‐responsive acrylic acid‐spiropyran hydrogel based on in situ acidity regulation. a) The conformational transition of the polyacrylic acid polymer in pH 4, and the chemical structures and net charge states of the merocyanine form (ODP) and spiropyran form (CDP) before and after irradiation with visible light. b) Plot of the characteristic pH change of ODP (0.5 mm) in water with an initial pH of 4, with light on and off. c) Schematic representation of the local contraction process of the PAAc‐spiropyran hydrogel under light stimulation.

Here, we report in‐situ stimuli transfer photo‐responsive acrylic acid‐spiropyran copolymer hydrogels that exhibit rapid and reversible shape‐morphing according to light irradiation in different environments. This rapid light response is attributed to the reversible photo‐responsive protonation of the spiropyran,^[^ ^27^ ^]^ which enables the in‐situ regulation of hydrogen bonding interactions within the polyacrylic acid network (**Figure** **1**). Importantly, the inner pH of PAAc hydrogel was tailored according to the sharp volume contraction/expansion behavior of polyacrylic acid near its p*K*_a_ (≈4) value,^[^ ^28^, ^29^ ^]^ so as to receive maximum amplitude in shape morphing. The photo‐responsive behavior in water‐deficient environments such as in air and in oil is realized by water transfer from one part of the hydrogel to another while keeping the net volume nearly constant. This strategy can be extended to include other spiropyran species with diverse structures. The reversible motion performances of programmable PAAc hydrogels are conveniently manipulated by controlling light, which sets an excellent example in utilizing PAAc‐based hydrogels for multi‐environment applications.

## 2 Results and Discussion

### 2.1 Fabrication and Characterization of PAAc‐Spiropyran Hydrogels

Polyacrylic acid (PAAc) hydrogel exhibits a sharp shrinking transition at pH 4 (Figure 1a), primarily attributed to changes in the COOH/COO^−^ ratio.^[^ ^30^ ^]^ Experimental findings further validate that the dissociation of carboxylic acid groups in PAAc hydrogel occurs around pH 4 by observing the zeta potential mutation (Figure S1, Supporting Information), consistent with the theoretical p*K*_a_ value of PAAc. To achieve in situ regulation of the internal acid environment within PAAc hydrogel, we synthesized a polymerizable water‐soluble spiropyran molecule (see Figures S2–S5, Supporting Information for details). As shown in Figure 1a, the ring‐opened merocyanine (ODP) molecule contains polymerizable acrylamide and quaternary ammonium groups, and it exhibits a water solubility of up to 52 g L^−1^. Upon irradiation, ODP undergoes isomerization and transforms into its ring‐closed spiropyran (CDP) form, resulting in the release of a proton and a decrease in net charge (from 2 to 1). The acidity changes in the aqueous solution (initial pH 4) during the isomerization process are studied (Figure S6a, Supporting Information). The concentration of hydrogen ions (H^+^) in the solution increases with increasing ODP concentration, reaching a peak at 0.5 mm. However, as the ODP content continues to increase, the concentration of released H^+^ starts to decline. Additionally, the color of the aqueous solution gradually deepens (Figure S6b, Supporting Information). Therefore, for the next experiments, a concentration of 0.5 mm for the ODP molecule is selected.

Figure 1b shows that during the isomerization process, the pH typically changes by 0.93 units under illumination. When the light is turned off, the pH rapidly returns to its initial value, indicating excellent cycle stability. The slightly higher pH variation in the first cycle may be due to the decomposition of a small amount of ODP molecules after initial light exposure. The UV–vis absorption spectra of the ODP and CDP in aqueous solution are shown in Figure S7 (Supporting Information). These molecules are covalently integrated into cross‐linked polymer networks through aqueous free‐radical polymerization. This process is in the presence of acrylic monomers, N,N'‐methylenebisacrylamide cross‐linkers, and ammonium persulfate initiators (Figure S8, Supporting Information). The pH of the solution is regulated by sodium hydroxide. The UV–vis absorption spectra of the hydrogels before and after irradiation are shown in Figure S9 (Supporting Information). The characteristic absorption peaks of ODP in the prepared hydrogel are difficult to detect due to the low ODP content (Figure S9b, Supporting Information). The prepared hydrogel samples exhibit high transparency, both before and after illumination (Figure S10, Supporting Information). According to our design illustrated in Figure 1c, we adjust the pH of the precursor solution to 4, causing the carboxylic acid groups in acrylic acid to dissociate. Upon illumination, ODP rapidly converts to CDP, releasing protons that regulate the local acidity of the hydrogel in situ, lowering the pH below the p*K*_a_ of polyacrylic acid. This enhances hydrogen bonding interactions within the hydrogel and, combined with increased hydrophobicity of the polymer backbone, leading to rapid and significant local contraction of the hydrogel. When the light is turned off, CDP moieties efficiently convert back to ODP by adsorbing protons in situ from the PAAc hydrogel. As a result, the initial pH of the hydrogel is restored, and it is expected to recover its initial shape.

### 2.2 Photo‐Stimuli Shape‐Morphing Mechanism of PAAc‐Spiropyran Hydrogels

**Figure 2:**
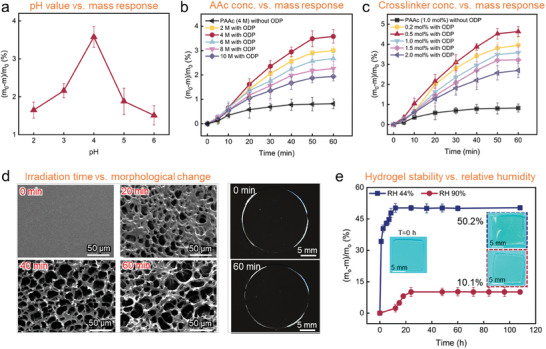
Light‐induced shape‐morphing mechanism of PAAc‐spiropyran hydrogels in air. a) Dependences of the degree of shape‐morphing of PAAc‐spiropyran gels on the pH of the pre‐gel sample. b) Water loss kinetics of hydrogel samples under light irradiation at different acrylic acid concentrations. c) Water loss kinetics of hydrogel samples under light irradiation at different crosslinker contents. d) SEM images showing phase evolution in 25 °C air for hydrogel irradiation for 60 min followed by liquid‐nitrogen quenching. After 60 min of light irradiation, the hydrogel still maintained a high level of transparency. e) The water loss of PAAc hydrogel in air over time under different relative humidity (RH) conditions. All experiments conducted in air with 90% RH using a light source with a wavelength of 405 nm and intensity of 100 mW cm^−2^. The values are mean values, and the error bars represent standard deviations of data collected from 5 tests of different separate samples.

We initially investigated the shape‐morphing performance of the PAAc‐spiropyran hydrogel at different pH values through measuring the mass change during light irradiation (**Figure** **2**a). As expected, the hydrogel shows the maximum light‐driven mass loss at a pH value of ≈4. However, the resulting water loss rate is only 3.5%. Similar phenomena of very low mass loss rates (3.5% and 4.6%, respectively) are observed in our experiments investigating the effects of monomer content and crosslinker content on the light response behavior (Figure 2b,c). This departure from the traditional pH response behavior observed in PAAc hydrogels with an abundance of water outside the hydrogel is significant (Figure S11, Supporting Information). In further experiments, we verify phase separation by monitoring the morphological evolution of the hydrogel over illumination time via SEM observation. The results are shown in Figure 2d. The initial state (0 min) corresponds to the non‐porous, homogeneous phase state before light irradiation. As irradiation went on, pore size increased. The gradually enlarging pore size is closely related to the decrease of crosslink density of the hydrogel network. In this specific situation, there are no factors other than phase separation that can cause the decrease of crosslink density. Therefore, we deduce that during irradiation, phase separation did occur. The persistent high transparency might be a result of the small scale of phase separation. The visual observation indicates the high transparency of hydrogel before and after irradiation. The persistent high transparency might be a result of small enough size of the separated phase which allows visible light to get pass through. Based on these results, we speculate that the stimuli‐responsive behavior of the PAAc‐spiropyran hydrogel is independent of the exchange of water in and out of hydrogel. Instead, it drives water to transfer from one part of the hydrogel to another, resulting in a nearly constant net volume. The hydrogels without ODP experience minor water loss under irradiation in a normal high‐humidity testing environment, which is attributed to unavoidable natural evaporation. Moreover, the local light absorption and structural changes in the hydrogels with ODP exacerbate the observed mass loss phenomenon. The photo‐responsive performance of the hydrogel initially increases and then decreases with increasing monomer or crosslinker concentration, peaking at 4 m and 0.5 mol% to AAc, respectively. The water retention capacity of a 1 cm × 1 cm × 0.5 mm square block hydrogel in air is investigated (Figure 2e). At 40% RH, the hydrogel lost 34.2% of its water mass within 1 h and reached a water loss equilibrium of 50.2% after 12 h, exhibiting noticeable dehydration. Under 90% RH, the hydrogel lost 2.3% of its water content after 12 h and reached a water loss equilibrium of 10.1% after 24 h. Importantly, the hydrogel maintained its moisture without affecting its photo‐responsive behavior. We demonstrate a simple method to prevent water loss in a moderate humidity environment by applying a layer of silicone oil (20 mPa s) to the surface of the hydrogel. This approach effectively retains water in a 40% RH environment (Figure S12, Supporting Information) while performing shape‐morphing without compromise (Figures S13 and S14, Supporting Information). Unless otherwise specified, our experiments are conducted under 90% RH.

### 2.3 Environmental‐Independent Reversal Shape‐Morphing of PAAc‐Spiropyran Hydrogels

**Figure 3:**
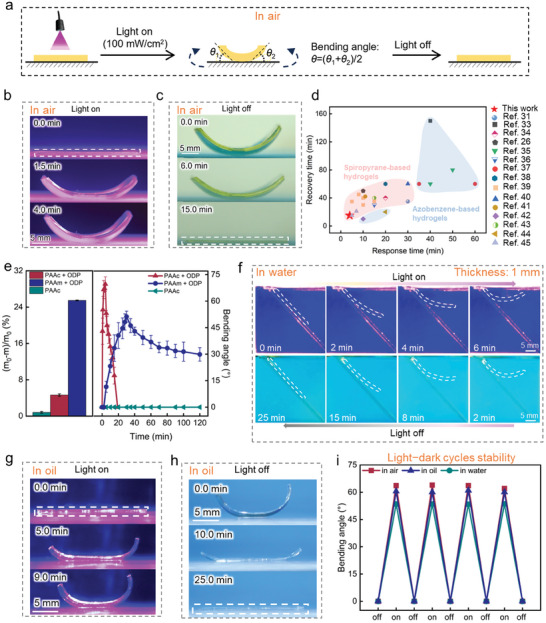
Reversal shape‐morphing of PAAc‐spiropyran hydrogel thin films in various environments. a) Schematic representation of bending deformation of strip‐shaped PAAc‐spiropyran hydrogel upon irradiation from above. The bending angle is defined as θ. b) Photographs of the bending process of PAAc‐spiropyran hydrogel in air under irradiation over time. c) Photographs of the recovery process of PAAc‐spiropyran hydrogel in air after the light is turned off. d) Comparing the properties of photo‐responsive hydrogels based on molecular photo‐switches. e) Water loss and shape‐morphing performance of hydrogels with different polymer backbones through light stimuli in air. f) Photographs of bending‐unbending processes of PAAc‐spiropyran hydrogel in water over time. g) Photographs of bending process of PAAc‐spiropyran hydrogel in dimethylsilicone oil (PMX‐200, 20 mPa s) under irradiation over time. h) Photographs of the recovery process of PAAc‐spiropyran hydrogel in dimethylsilicone oil (PMX‐200, 20 mPa s) after the light is turned off. i) Plot of changes in maximum bending angles over four light‐dark cycles for PAAc‐spiropyran hydrogel in various environments. The values are mean values, and the error bars represent standard deviations of data collected from 5 tests of different separate samples.

In our design, as a homogenous constitute, the phototactic artificial network shape morphs under light irradiation (**Figure** **3**a). The strip‐shaped PAAc‐spiropyran hydrogel thin film (0.5 mm thick) bends toward the light source in the air due to inhomogeneous contraction within the hydrogel. Namely, the network contraction only happens in part of the material (perhaps as a result of light absorption of ODP moieties), driving water to diffuse from one part to another, and retaining the net volume nearly constant.

When illuminated in an air environment, the PAAc‐spiropyran hydrogel rapidly reaches equilibrium within 4 min, achieving a bending angle of 69.6° (Figure 3b; Figure S15, Supporting Information). Conversely, when the light is turned off, the hydrogel quickly restores its original shape within 15 min (Figure 3c; Movies S1 and S2, Supporting Information). Notably, thinner hydrogels have shorter response and recovery times. For example, when the hydrogel thickness is 0.2 mm, it reaches the maximum bending angle (≈73.6°) and recovers within 3 and 7 min, respectively (Figure S15, Supporting Information). Compared to other reported molecular photoswitches‐based hydrogels with the same hydrogel thickness,^[^ ^26^, ^31^, ^32^, ^33^, ^34^, ^35^, ^36^, ^37^, ^38^, ^39^, ^40^, ^41^, ^42^, ^43^, ^44^ ^]^ our hydrogel exhibits superior shape‐morphing performance in terms of response time, and recovery time (Figure 3d). The influence of light intensity on the shape‐morphing behavior of the hydrogel is illustrated in Figure S16 (Supporting Information). At a light intensity of 100 mW cm^−2^, both the bending angle and response time of the hydrogel reach their maximum. Further increasing the light intensity does not significantly enhance the photo‐responsive capability. In addition, we prepared hydrogels with varying ODP concentrations to test their light‐responsive behaviors. The results, shown in Figure S17 (Supporting Information), align with our results, the hydrogel with a 0.5 mm ODP monomer concentration exhibits the best shape‐morphing capability. Figure 3e examines the rapid and reversible shape‐morphing mechanism of PAAc‐spiropyran hydrogel in air by analyzing the water loss and photo‐responsive behavior of hydrogels with different polymer backbones under irradiation. The significant difference in water loss rates between the PAAc‐spiropyran hydrogel and the PAAm‐spiropyran and PAAc hydrogel (4.6%, 25.5%, 0.8%, respectively) further confirms the independent water environment shape‐morphing mechanism of PAAc‐spiropyran hydrogel. The higher mass loss in the case of PAAm‐spiropyran indicates a typical water exclusion effect of spiropyran.^[^ ^31^ ^]^ Additionally, compared with PAAc‐spiropyran hydrogel, the PAAm‐spiropyran hydrogel exhibits a slow photo‐response time, limited bending angle, and irreversible recovery ability in an air environment. In contrast, PAAc hydrogels, which lack ODP groups, do not exhibit any light‐responsive behavior in air. This highlights the crucial role of extensive reversible hydrogen bonding interactions triggered by the ODP isomerization process in PAAc‐spiropyran hydrogels for their exceptional shape‐morphing performance in a water‐free environment.

The shape‐morphing behavior of the PAAc‐spiropyran hydrogel is believed to be applicable in all environments. As shown in Figure 3f and Figure S18 (Supporting Information), one side of a strip‐shaped PAAc‐spiropyran hydrogel thin film (1 mm thick) is affixed to a glass plate in a water environment. Light is then directed onto the unfixed part at the other end. The hydrogel demonstrated impressive phototactic bending behavior, achieving the maximum bending angle within 6 min. After the light is turned off, the hydrogel returns to its original state within 25 min (Movies S3 and S4, Supporting Information). Note that the hydrogel strip has reached swelling equilibrium by immersion in deionized water overnight (>12 h) prior to the light stimulation, and the entire experiment is also carried out in deionized water. The non‐sabotaged photo‐responsiveness in our case clearly indicates that the weak acidic environment within the hydrogel remains stable underwater, which can be understood by the weak electrolyte nature of the main component, namely the carboxyl groups on acrylic acid. Since these carboxyl groups are chemically attached to the inside of the polymer network, the weak acidic condition cannot be further diluted by the vast water outside, giving a stable weak acidic environment to the hydrogel.

In the oil environment, this hydrogel also exhibits excellent photo‐stimuli shape‐morphing behaviors (Figure 3g,h), with the maximum bending angle (≈66.2°) achieved within 9 min and recovery within 25 min (Figure S19, Supporting Information). Figure 3i shows the recovery of the maximum bending angle after four bending‐unbending cycles in various environments, demonstrating the good cyclic ability of this artificial network. We observed that as the number of cycles further increased, the bending angle gradually decreased, especially after 6 cycles (Figure S20, Supporting Information). This may be attributed to the instability of the photo‐responsive molecules.^[^ ^45^ ^]^ Selecting a system with improved stability will be a key focus in our future work. It is worth noting that the phototactic response behavior of the hydrogel is independent of the substrate and unaffected by its placement. In Figure S21 (Supporting Information), when a strip‐like PAAc‐spiropyran hydrogel is sequentially illuminated from the left side, it bends left and reverts to its original state when the light is turned off. Similarly, when illuminated from the right side, it bends right (Figure S22, Supporting Information). In addition, we synthesize the spiropyran‐based photoacid **1** following literature reports,^[^ ^46^ ^]^ and incorporate the photoacid **1** into the polyacrylic acid hydrogel through physical mixing. The newly prepared photoacid **1**‐based PAAc hydrogel also demonstrates outstanding photo‐responsive behavior (Figure S23, Supporting Information). It achieves a rapid bending of up to a maximum angle of 60° within just 5 min in the air and returns to its initial state in a mere 16 min (Figure S24, Supporting Information). These results provide evidence for the universality of this strategy.

### 2.4 Reversible Actuation Performances of PAAc‐Spiropyran Hydrogels Under Light

**Figure 4:**
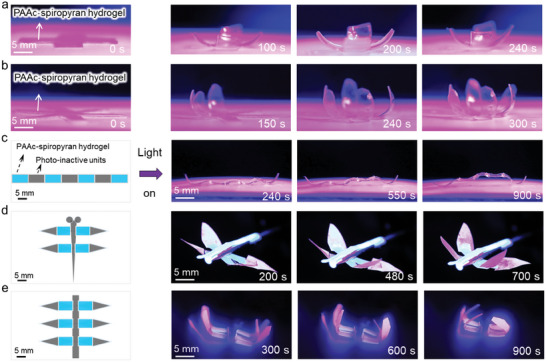
Reversible actuation of programmable PAAc‐spiropyran hydrogel in the air triggered by light. a,b) Bending configurations of the four‐petal and six‐petal flower‐shaped PAAc‐spiropyran hydrogel films by light irradiation from the top, respectively. c) Linear block structure containing four PAAc‐spiropyran hydrogel units (blue) and four photo‐inactive units (gray) undergo shape transformations upon light irradiation; d) Dragonfly‐shaped structure containing four PAAc‐spiropyran (blue) as a hinge component in assembled devices driving the photo‐inactive units (gray) to bend upon light irradiation. e) gripper‐like model containing six PAAc‐spiropyran units (blue) as a hinge component in assembled devices driving the bending of the photo‐inactive units (gray) upon light irradiation. All experiments are conducted using light with a wavelength of 405 nm and an intensity of 100 mW cm^−2^. The PAAc‐spiropyran hydrogels have a thickness of 0.5 mm.

The impressive shape‐morphing capabilities of the PAAc‐spiropyran hydrogel in various environments have motivated us to utilize it as a photo‐active unit for constructing functional assembled structures capable of programmable shape changes in response to light. **Figure** **4**a,b depict thin films (0.5 mm thick) of PAAc‐spiropyran hydrogels, which initially resemble four‐petal and six‐petal flat flower shapes. When irradiated from above, these hydrogels exhibit phototactic bending behaviors, resembling the shape changes observed in the natural phenomenon of flower blooming in the air (Movies S5–S8, Supporting Information). Notably, they demonstrate ultrafast bending abilities, completing the bending process in 240 and 300 s, respectively. Importantly, these photo‐actuator behaviors are highly reversible, with the PAAc‐spiropyran hydrogels bending and unbending upon switching the light on and off. They fully recover to their original states within only 15 min in the air (Figures S25 and S26, Supporting Information). Furthermore, we have designed more complex assembled structures by combining photo‐active and photo‐inactive units. Figure 4c shows a linear structure with a specific sequence of four photo‐active and four photo‐inactive units. In the dark, the structure remains flat. However, upon light irradiation in air, it exhibits wave‐like motion and can lift the photo‐inactive components (PAAc hydrogel), resembling a lifting robot. This programmability enables us to use the PAAc‐spiropyran hydrogel as a hinge component in assembled devices that drive the motion of nonphotoactive units. Figure 4d illustrates a dragonfly‐shaped structure with four photo‐active units as hinges, which drive the bending of the photo‐inactive units under irradiation, mimicking the movement of a flying dragonfly. This experiment also confirms that the exceptional actuating behavior of the PAAc‐spiropyran hydrogel is not due to asymmetric interactions from the solid substrate material and environmental factors. Instead, it is caused by the inhomogeneous contraction within the hydrogel itself. Figure 4e presents a gripper‐like model comprising six photo‐active units as hinges, which drive the bending of the photo‐inactive units through irradiation. These results further validate the feasibility of developing a highly adaptive and intelligent photo‐induced motion functionality by utilizing the specific structure and geometric shape of our PAAc‐spiropyran hydrogel in various environments.

## 3 Conclusion

In summary, we have successfully prepared a rapid light‐responsive shape‐morphing hydrogel based on poly(spiropyran‐co‐AAc). The as‐prepared hydrogel morphs its shape in multi‐environment, without relying on significant water uptake or release. Results show that the contraction of the hydrogel network happens in part of the material upon light irradiation, driving water transfer from one part to another, while the remaining net volume of the gel unchanged. This unique shape‐morphing manner enhances the adaptability of hydrogel to different environments. Furthermore, we have developed an innovative in‐situ stimuli transfer mechanism that enables faster and environment‐independent stimuli‐responsiveness in hydrogels. This mechanism involves reversible photo‐responsive protonation of ODP, which effectively regulates the local hydrogen bonding network of polyacrylic acid. This strategy is flexible and can be adopted in various spiropyran species carrying different structures. The PAAc‐spiropyran hydrogel, whether in its standalone form or as a hinge component in assembled devices, exhibits remarkable actuation performances. We would like to reiterate that the new mechanism helps in decoupling the materials from their external water environments. Therefore, our current study improves the environment adaptability of stimuli‐responsive hydrogel in fields such as soft robotics, biomaterials, smart devices, and many more.

## 4 Experimental Section

### Synthesis of ODP

The ODP molecule was prepared according to a previously reported method.^[^ ^47^ ^]^ Specifically, 0.02 m of 2,3,3‐trimethylindolenine and 0.024 m of 1, 3‐Dibromopropane were thoroughly stirred in a 50 mL acetonitrile solution. Nitrogen was then injected into the reaction container to remove the oxygen present. The mixture was refluxed for 24 h at 82 °C under a nitrogen environment. After that, cooled to room temperature. The resulting product was filtered, washed with a significant amount of ethyl acetate solution, collected as a solid, and dried in a vacuum drying oven to obtain compound **2**. ^1^H NMR (400 MHz, DMSO‐d_6_, δ): 7.85 (m, 2H), 7.62 (m, 2H), 4.30 (s, 2H), 3.49 (d, J = 16 Hz, 2H), 2.13 (m, 3H), 1.96 (m, 3H), 1.55 (s, 6H).

The 2 mm of compound **2** and 2.4 mm of salicylaldehyde were thoroughly mixed in ethanol under a nitrogen flow environment. The mixture was then heated gradually to 80 °C and maintained at this temperature for 12 h under nitrogen protection. Once the reaction was complete, the mixture was cooled to room temperature, filtered, and washed with ethyl acetate. The filter cake was collected, and the resulting orange compound **3** was obtained after vacuum drying. ^1^H NMR (400 MHz, DMSO‐d_6_, δ): 10.71 (s, 1H), 8.31 (s, 1H), 8.89 (s, 2H), 8.32‐8.23 (m, 8H), 7.73‐7.57 (m, 4H), 7.43 (t, J = 12 Hz, 1H), 7.10‐6.93 (m, 2H), 4.41 (t, J = 8 Hz, 2H), 2.97 (t, J = 8 Hz, 2H), 2.18 (t, J = 8 Hz, 2H), 1.81 (s, 6H).

Compound **3** and N, N‐Dimethylaminopropyl acrylamide (DMAPAAm) were dissolved in DMF in equimolar proportions. The reaction mixture was stirred at room temperature for 24 h. The solvent was then removed through vacuum distillation to obtain the crude product of ODP. The crude product was purified by column chromatography (silica gel) using MeOH: DCM (1:40, v/v) as eluent to obtain ODP, yield 70% of brown solid. ^1^H NMR (400 MHz, DMSO‐d_6_, δ): 7.93 (s, 1H), 7.14‐7.02 (m, 3H), 6.97 (d, J = 4 Hz, 1H), 6.87 (t, J = 4 Hz, 1H), 6.80 (d, J = 8 Hz, 1H), 6.71‐6.65 (m, 2H), 6.54 (s, 1H), 6.20 (m, 1H), 6.11 (m, 1H), 5.61 (m, 1H), 3.31‐3.15 (m, 3H), 3.01 (d, J = 4 Hz, 1H), 2.94 (t, J = 8 Hz, 2H), 2.88 (s, 3H), 2.72 (s, 3H), 2.69 (s, 5H), 2.65 (s, 1H), 1.77 (m, 3H),1.21 (s, 3H), 1.10 (s, 3H). ESI‐MS: m/z calculated for C_29_H_39_O_2_N_3_ 461.30; [M + H]^+^, 461.30; found: 462.30.

### Preparation of PAAc‐Spiropyran Hydrogel

Take the 4 m AAc as an example. For 2.88 g of acrylic acid (AAc, 4 m), 30.83 mg of N, N‐methylenebisacrylamide (MBAA) at a concentration of 0.5 mol% relative to the monomer, and 2.3 mg ODP monomer at a concentration of 0.5 mm were added to 10 mL of deionized water. The mixture was dissolved and thoroughly stirred. Next, 31.95 mg of ammonium persulfate (APS) at a concentration of 0.35 mol% relative to the monomer and 20 µL of N, N, N', N'‐tetramethylethylenediamine (TEMED) at a concentration of 0.34 mol% relative to the monomer were added to the mixture. To adjust the pH of the gel precursor solution to 4, 1.58 g of sodium hydroxide (NaOH) at a concentration of 16 m was added. The precursor solution was then poured into a glass mold and heated at 65 °C in an oven for 2 h to complete the thermal polymerization and obtain the final hydrogel. Circular hydrogels of different diameters were obtained using a metal hole puncher.

### Preparation of PAAm‐Spiropyran Hydrogel

For the synthesis of the hydrogel, 2.84 g of acrylamide (AAm, 4 m) was dissolved in 10 mL of deionized water. Then, 2.3 mg of ODP monomer was added. N, N‐methylenebisacrylamide (0.5 mol% relative to monomer concentration, 30.83 mg), ammonium persulfate (0.35 mol% relative to monomer concentration, 31.95 mg), and tetramethylethylenediamine (0.34 mol% relative to monomer concentration, 20 µL) were dissolved and mixed evenly. The pH of the pregel solution was adjusted to four using a 16 m sodium hydroxide solution. The pregel solution was poured into a glass mold and heated at 65 °C in an oven for 2 h to obtain the hydrogel. Different diameters of round hydrogels were obtained using a metal punch.

### Photo‐Response test of PAAc‐Spiropyran Hydrogel in Various Environments

Take the oil environment as an example. The hydrogel was immersed in dimethylsilicone oil (PMX‐200, 20 mPa s) and subjected to a light response experiment in a 90% humidity environment. The light was irradiated from above (405 nm, 100 mW cm^−2^). After reaching the maximum bending angle, turn off the light, and the recovery process is recorded Then image was used to measure the change of bending angle with time.

### Cycle Experiments of PAAc‐Spiropyran Hydrogel in Various Environments

The hydrogel was prepared and cut into strip structures measuring 2 cm × 1 cm × 0.5 mm in length, width, and thickness, respectively. In an environment with 90% humidity, the hydrogel was exposed to visible light with a wavelength of 405 nm and an intensity of 100 mW cm^−2^. The light was irradiated from the upper end of the sample, and the entire process was recorded using a camera. When the bending angle reached its maximum, the light was turned off to allow for recovery. This process was repeated to test the cycling ability, with a maximum light exposure duration of 15 min and a dark period of 50 min.

In the stability experiment under a water environment, the prepared hydrogel with initial dimensions of 1 cm × 1 cm × 0.5 mm was swelling in deionized water. After achieving swelling equilibrium, the sample was cut into a hydrogel strip measuring 2 cm × 1 cm × 1 mm in length, width, and thickness, respectively. One end of the hydrogel is glued to a glass plate, while the other end is allowed to drop naturally. The glass plate is then placed in water, and light is shone directly above the water surface and at the tail of the gel. The light used has a wavelength of 405 nm and an intensity of 100 mW cm^−2^. The entire process is recorded using a camera, and when the bending angle reaches its maximum, the lights are turned off and the hydrogel is allowed to recover in the dark. This process is repeated to test the cycle performance of underwater. The duration of light exposure can reach up to 20 min, followed by a dark placement period of up to 80 min.

The cycle performance tests in oil environment were conducted in a similar manner to those in the air. The hydrogel samples were exposed to light for a duration of 30 min, followed by a dark standing time of 90 min.

### Water Loss Experiments of Hydrogels with Different Polymer Networks

Using the PAAc‐spiropyran sample as an example, the prepared hydrogel was placed in neutral water to swell for 27 h. After removing it, excess water on the surface was gently wiped with filter paper, and then it was cut into a cylinder with a diameter of 1 cm, and its mass was defined as m0. The light response experiment was then conducted in an air humidity of 90%, and the mass of the hydrogel was measured at different times of illumination, defined as m. The mass loss of the hydrogel was calculated using the formula (m_0_ – m)/m_0_. The experimental method for the polyacrylamide‐spiropyran hydrogel samples followed the same procedure as for the PAAc‐spiropyran samples. The pure polyacrylic hydrogel was tested by swelling in a pH 4 solution for 24 h, wiping the excess water on the surface with filter paper, and then it was cut into a cylinder with a diameter of 1 cm, and measuring the initial mass m_0_. It was then submerged in water with a pH of 3, and left to stand for 24 h, and the final mass m is measured in the same way.

### Scanning Electron Microscopic (SEM) Test

In this experiment, humidity was maintained at 90%, with the light source from above (405 nm, 100 mW cm^−^^2^). Samples were irradiated for different durations before freeze‐drying. The morphological changes of PAAc‐spiropyran hydrogels were observed using scanning electron microscopy (SEM).

## Conflict of Interest

The authors declare no conflict of interest.

## Author Contributions

L.L.Z., J.J., and X.Y. equally contributed to this work. Y.X., L.L.Z., L.W.W., and Y.M.L. performed the methodology. L.W.W., Y.M.L., W.P.Y., Z.J.L., C.P.L., and X.M.Y. performed investigation. X.Y., L.L.Z., L.W.W., J.J., Y.M.L., W.P.Y., Z.J.L., and C.P.L. performed formal analysis. L.L.Z., J.J., and X.Y. performed resources. L.L.Z. and X.Y. performed supervision and wrote the original draft. X.Y., L.L.Z., L.W.W., Y.M.L., W.P.Y., Z.J.L., and J.J. wrote, reviewed, and edited the original draft.

## Supporting information



Supporting Information

Supplemental Movie 1

Supplemental Movie 2

Supplemental Movie 3

Supplemental Movie 4

Supplemental Movie 5

Supplemental Movie 6

Supplemental Movie 7

Supplemental Movie 8

## Data Availability

The data that support the findings of this study are available in the supplementary material of this article.
