# Supplementary material for: In Situ Stimuli Transfer in Multi‐Environment Shape‐Morphing Hydrogels Based on the Copolymer Between Spiropyran and Acrylic Acid
Source: Adv Sci (Weinh). 2025 Mar 7;12(17):2416173. doi: 10.1002/advs.202416173 (PMC12061295; doi:10.1002/advs.202416173)
Supplement: Supplementary file 1 — Supporting Information [file ADVS-12-2416173-s002.docx]

Supporting Information

In-situ Stimuli Transfer in Multi-environment Shape-morphing Hydrogels Based on the Copolymer Between Spiropyran and Acrylic Acid

Liwei Wu, Yiming Liu, Wenpei Yang, Zejun Liu, Cuiping Liu, Xiaomin Yuan, Lingling Zhang*, Jie Ju*, Xi Yao*

**1. Materials**

2,3,3-trimethylindolenine (98%, Adamas-beta), 1,3-dibromopropane (99%, Aladdin), Salicylaldehyde (99%, Adamas-beta), dimethylaminopropylacrylamide (DMAPAAm, > 97%, stabilized with MEHQ, Aladdin), acetonitrile (CH_3_CN, 99.0%, McLean), ethanol (EtOH, Anhui ante Co., LTD), dimethylformamide (DMF, Tianjin Kemiou Chemical Reagent Co., LTD.), Ethyl acetate (McLean), acrylic acid (AAc, 98%, MERYER), N,N'-Methylenebisacrylamide (MBAA, 99%, J&K), ammonium persulfate (APS, 98%, Sigma Aldrich), N,N,N',N'-tetramethylene ethylenediamine (TEMED, 99%, McLean), Sodium hydroxide (NaOH, General-reagent®), Hydrochloric acid (HCl, General-reagent®). Photoacid **1** was synthesized by a procedure according to previously reported method.^[1]^ All reagents were used as received without further purification. Deionized water was used for all the experiments.

**2. Supporting Figures**

**Figure S1.** The zeta potential of polyacrylic acid hydrogel samples at different pH levels was measured at a monomer concentration of 4M.

**Figure S2.** The synthetic route for the ODP molecule.

**Figure S3.** The ^1^H NMR of compound **2**.

**Figure S4.** The ^1^H NMR of compound **3**.

**Figure S5.** The ^1^H NMR of compound ODP.


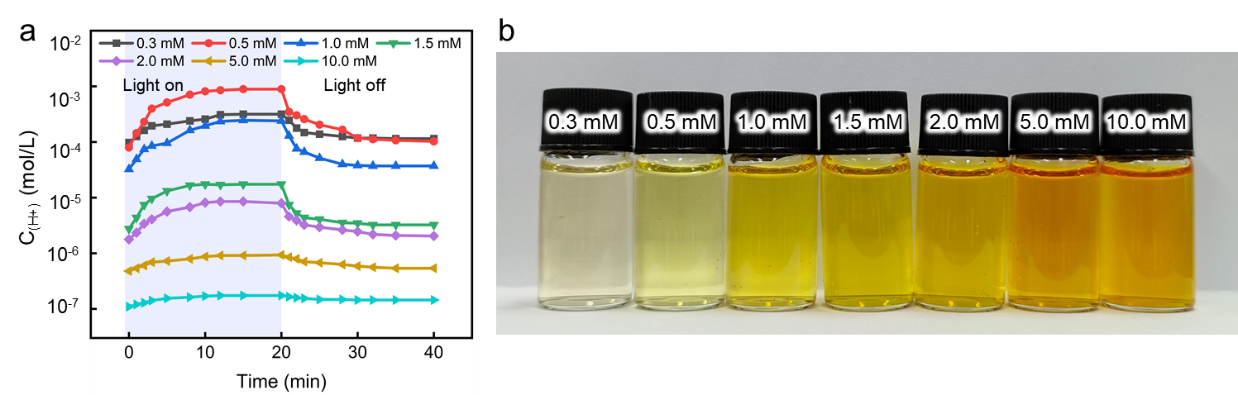


**Figure S6.** The proton-releasing properties of ODP molecules under light conditions. (a) Kinetic curves of proton concentration released over time under irradiation during the isomerization of various ODP concentrations. (b) Image of color change of ODP aqueous solution during isomerization.


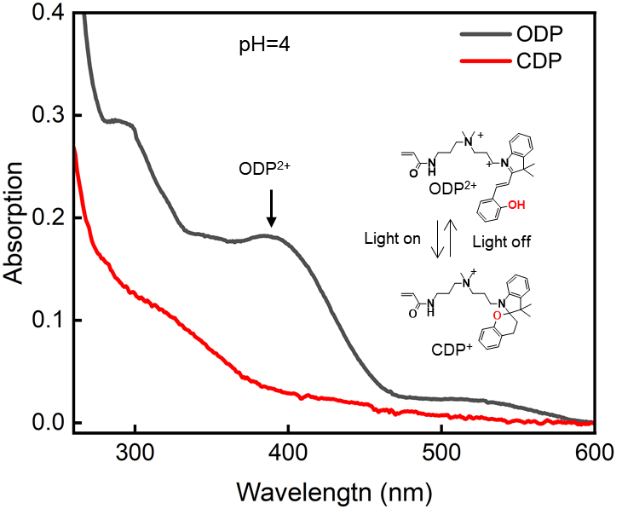


**Figure S7.** The UV-Vis absorption spectra of the ODP and CDP in aqueous solution (0.5 mM).


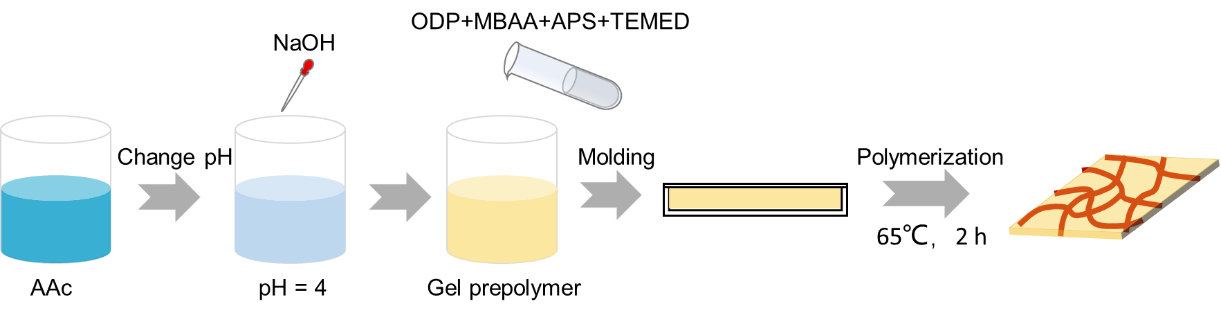


**Figure S8.** Scheme of the preparation procedure of PAAc-spiropyran hydrogel.


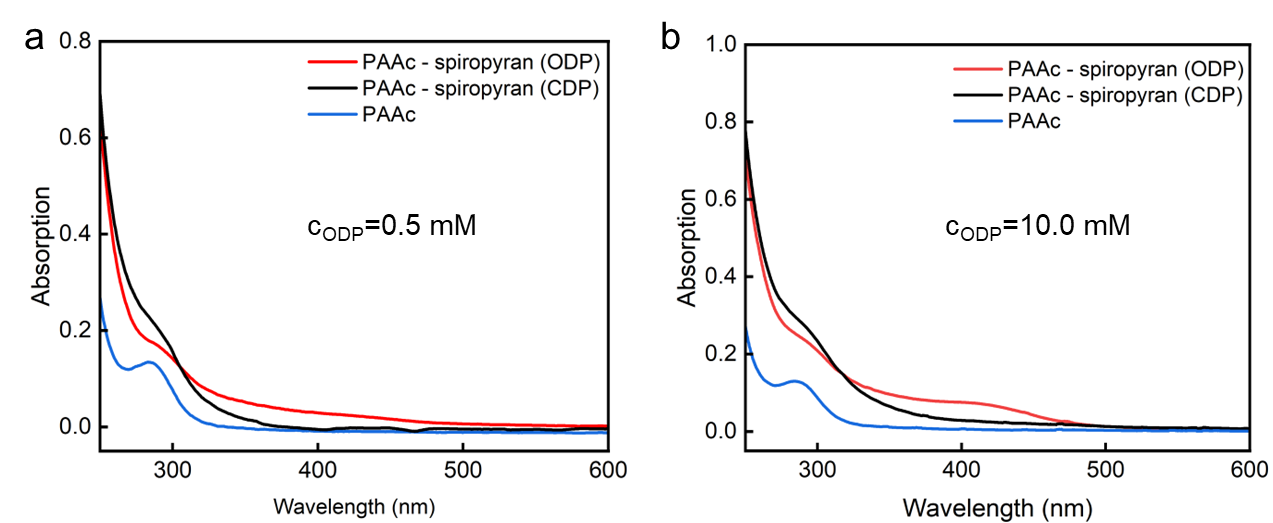


**Figure S9.** UV-visible absorption spectra of PAAc-spiropyran hydrogels before and after irradiation. At low ODP content, the characteristic absorption peak of ODP is not easily detectable in the UV absorption spectrum (a). However, as the ODP content increases, the characteristic peak become clearly observable (b).


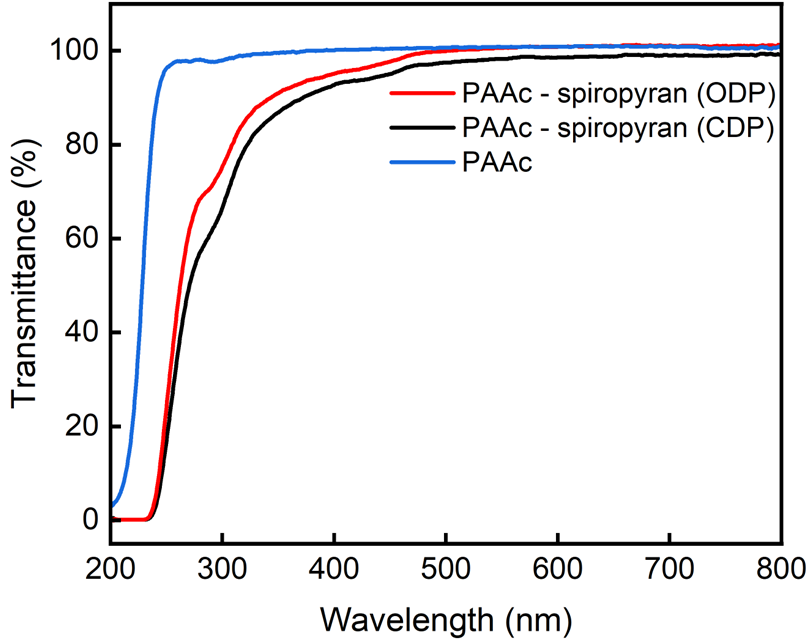


**Figure S10.** Transparency of PAAc and PAAc-spiropyran hydrogels before and after irradiation.


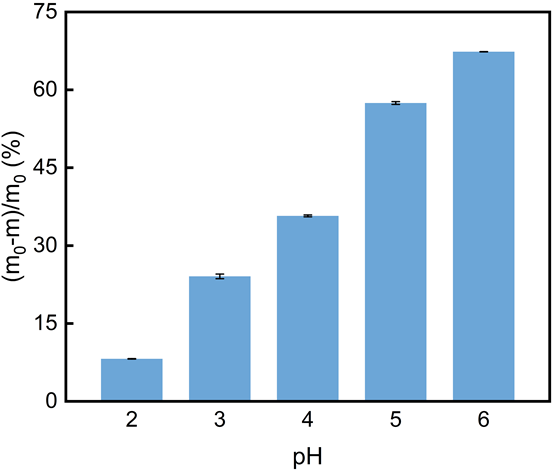


**Figure S11**. The mass loss rate of pH-responsive PAAc hydrogel. The water loss rate of polyacrylic acid hydrogels with different initial pH values. The experiment involved measuring the water loss rate of polyacrylic acid hydrogels when soaked in an aqueous solution with a pH less than one unit lower. The hydrogel underwent swelling equilibrium in the corresponding pH aqueous solution, followed by wiping off the surface water and weighing its initial mass (m_0_). Subsequently, it was immersed in an aqueous solution with a lower pH than its initial pH. For instance, a PAAc hydrogel with a pH of 4 was immersed in an aqueous solution with a pH of 3 to achieve swelling equilibrium, and the resulting mass (m) after equilibrium was measured using the same method.


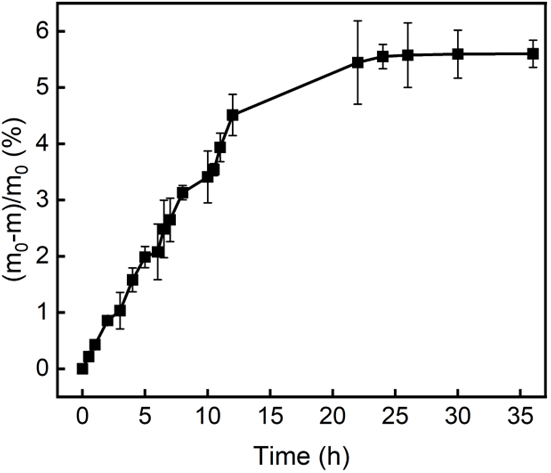


**Figure S12.** The water loss of PAAc-spiropyran hydrogel coated with a layer of silicone oil (20 mPa s) in air over time under 40% relative humidity (RH) conditions.


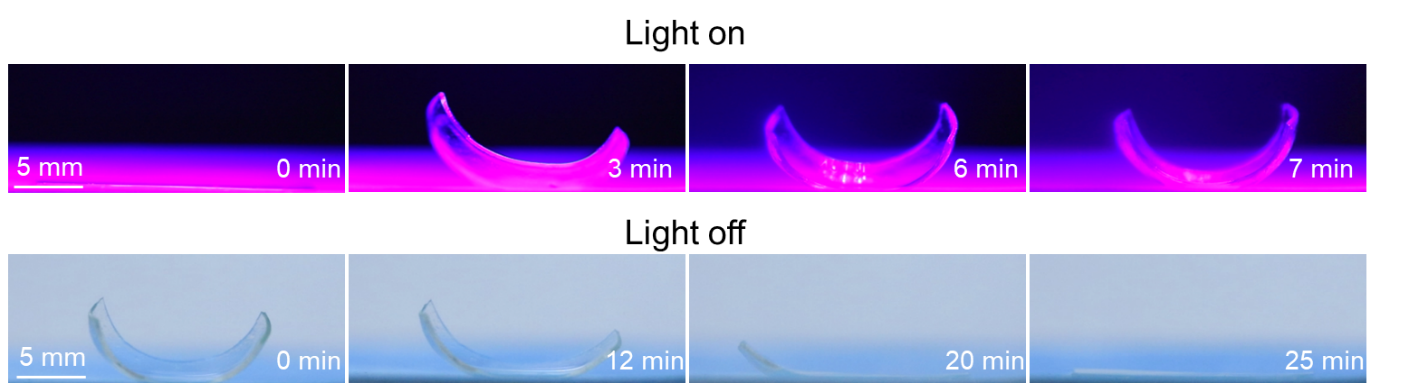


**Figure S13.** Photographs of bending-unbending processes of PAAc-spiropyran hydrogel coated with silicone oil (20 mPa s) in air over time under 40% relative humidity (RH) conditions.


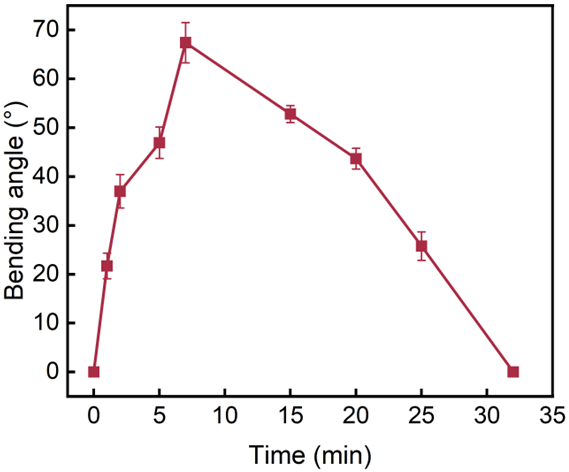


**Figure S14.** Plot of bending angles as a function of irradiation time in PAAc-spiropyran hydrogels coated with silicone oil (20 mPa s) in air over time under 40% relative humidity (RH) conditions.

**Figure S15.** Plot of bending angles as a function of irradiation time in PAAc-spiropyran hydrogels with different thickness.

**Figure S16.** The relationship between light intensity and shape-morphing performance of PAAc-spiropyran hydrogel under different light intensity.


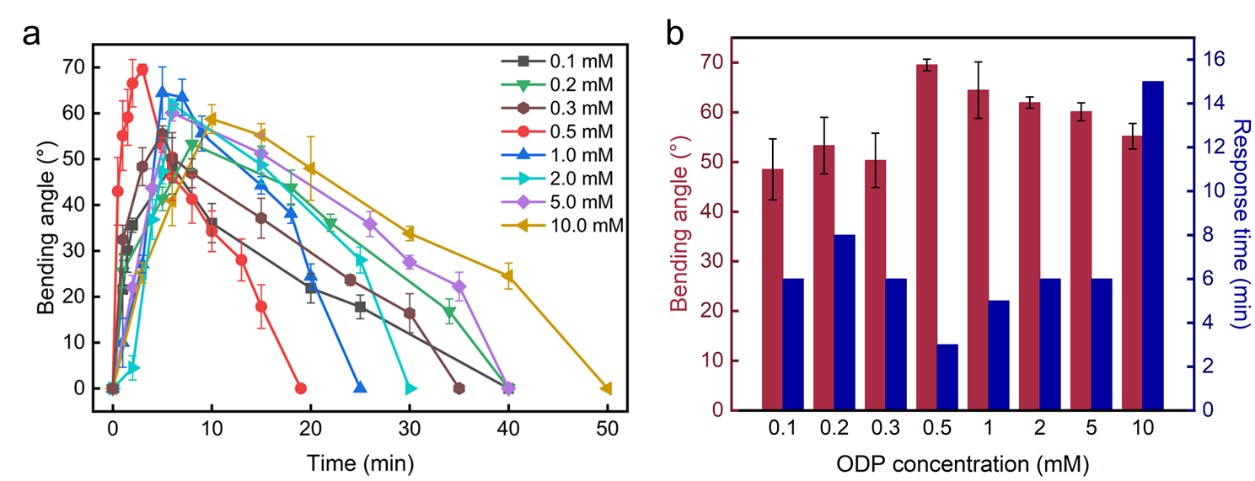


**Figure S17.** (a) Plot of bending angles as a function of irradiation time in PAAc-spiropyran hydrogels with ODP concentration. (b) The maximum bending angle and response time vary with the concentration of ODP.


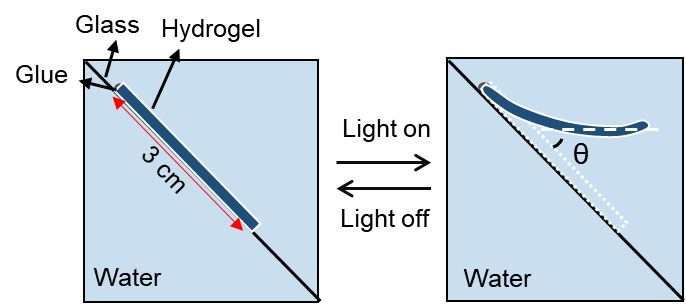


**Figure S18.** Schematic representation of bending deformation of strip-shaped PAAc-spiropyran hydrogel upon irradiation from above under water. The bending angle is defined as θ.


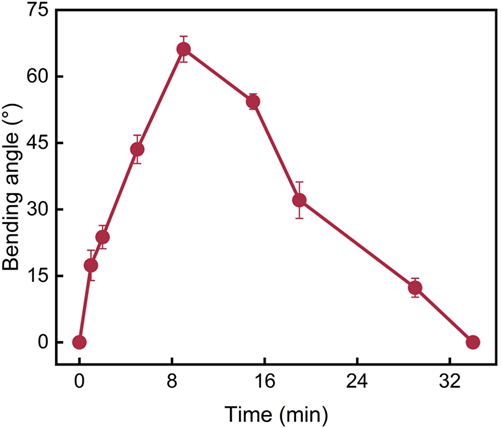


**Figure S19.** Plot of bending angles as a function of irradiation time in PAAc-spiropyran hydrogels in oil (PMX-200, 20 mPa s) environment.

**Figure S20.** Plot of changes in maximum bending angles over ten light-dark cycles for PAAc-spiropyran hydrogel in various environments.

**Figure S21.** The phototactic response behavior of stip-like PAAc-spiropyran hydrogel in air. (a) Photographs of bending process of PAAc-spiropyran hydrogel in air from left irradiation over time. (b) photographs of recovery process of PAAc-spiropyran hydrogel in air after the light is turned off.

**Figure S22.** Photographs of phototactic bending process of PAAc-spiropyran hydrogel in air from right irradiation over time.

**
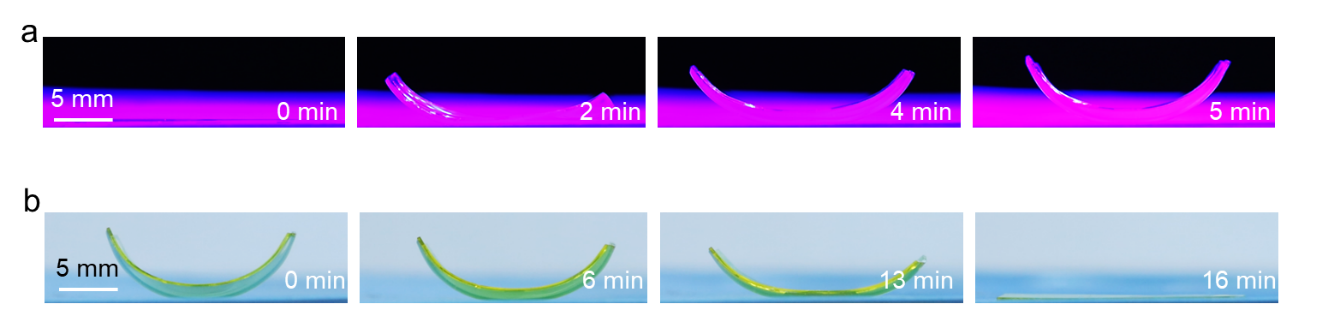
**

**Figure S23.** The photo-response of photoacid **1**-based PAAc hydrogel in air. (a) Photographs of bending process of photoacid **1**-based hydrogel in air under irradiation over time. And (b) photographs of recovery process of PAAc-spiropyran hydrogel in air after the light is turned off.


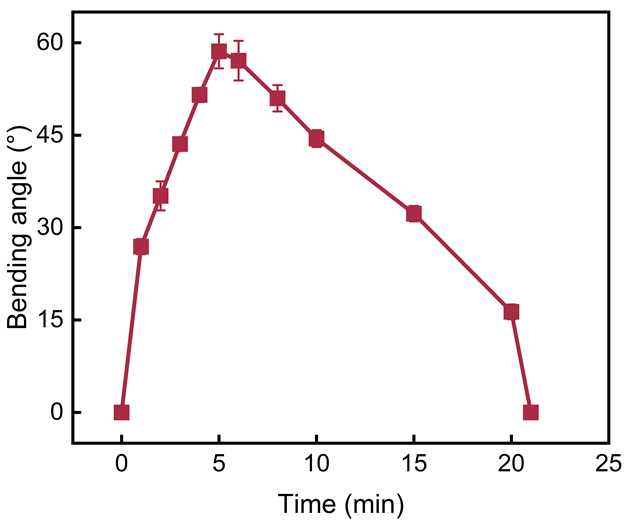


**Figure S24.** Plot of bending angles as a function of irradiation time in photoacid **1**-based hydrogel in air under irradiation over time.


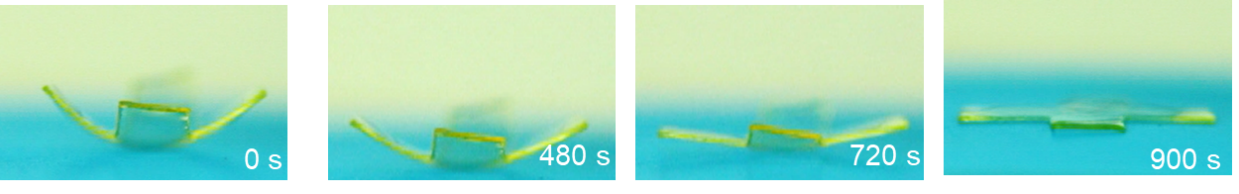


**Figure S25.** Photographs of recovery process of four-petal follower-shaped PAAc-spiropyran hydrogel in air after the light is turned off.

**
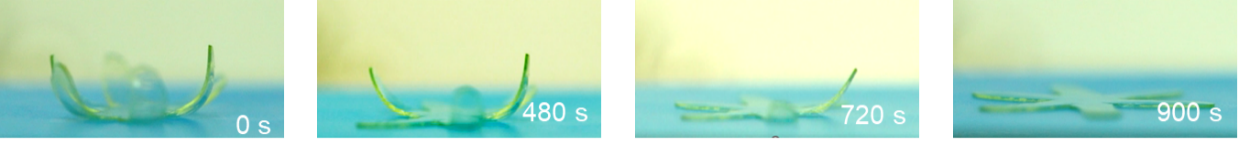
**

**Figure S26.** Photographs of recovery process of six-petal follower-shaped PAAc-spiropyran hydrogel in air after the light is turned off.

**3. Supporting movies**

**Movie S1:** The strip-like PAAc-spiropyran hydrogel exhibits shape-morphing behavior in air under irradiation.

**Movie S2:** The recovery process of strip-like PAAc-spiropyran hydrogel in air after the light is turned off.

**Movie S3:**  strip-like PAAc-spiropyran hydrogel exhibits shape-morphing behavior underwater under irradiation.

**Movie S4:** The recovery process of strip-like PAAc-spiropyran hydrogel underwater after the light is turned off.

**Movie S5:** The four-petal flower-shaped PAAc-spiropyran hydrogel films exhibit phototactic bending behavior when irradiated from the top in air.

**Movie S6:** The recovery process of four-petal flower-shaped PAAc-spiropyran hydrogel films in air after the light is turned off.

**Movie S7:** The six-petal flower-shaped PAAc-spiropyran hydrogel films exhibit phototactic bending behavior when irradiated from the top in air.

**Movie S8:** The recovery process of six-petal flower-shaped PAAc-spiropyran hydrogel films in air after the light is turned off.

**4. Reference**

[1] Z. Shi, P. Peng, D. Strohecker, Y. Liao, *J. Am. Chem. Soc.* **2011**, *133*, 14699-14703.
